# Water-soluble chlorophyll-binding proteins from *Arabidopsis thaliana* and *Raphanus sativus* target the endoplasmic reticulum body

**DOI:** 10.1186/s13104-015-1333-3

**Published:** 2015-08-20

**Authors:** Shigekazu Takahashi, Kyoko Aizawa, Katsumi Nakayama, Hiroyuki Satoh

**Affiliations:** Department of Biomolecular Science, Faculty of Science, Toho University, 2-2-1 Miyama, Funabashi, Chiba, 274-8510 Japan

**Keywords:** Chlorophyll, ER body, Water-soluble chlorophyll-binding protein, WSCP

## Abstract

**Background:**

Non-photosynthetic chlorophyll (Chl) proteins called water-soluble Chl-binding proteins are distributed in Brassicaceae plants. *Brassica oleracea* WSCP (BoWSCP) and *Lepidium virginicum* WSCP (LvWSCP) are highly expressed in leaves and stems, while *Arabidopsis thaliana* WSCP (AtWSCP) and *Raphanus sativus* WSCP (RshWSCP) are highly transcribed in floral organs. BoWSCP and LvWSCP exist in the endoplasmic reticulum (ER) body. However, the subcellular localization of AtWSCP and RshWSCP is still unclear. To determine the subcellular localization of these WSCPs, we constructed transgenic plants expressing Venus-fused AtWSCP or RshWSCP.

**Results:**

Open reading frames corresponding to full-length AtWSCP and RshWSCP were cloned and ligated between the cauliflower mosaic virus 35S promoter and *Venus*, a gene encoding a yellow fluorescent protein. We introduced the constructs into *A. thaliana* by the floral dip method. We succeeded in constructing a number of transformants expressing Venus-fused chimeric AtWSCP (AtWSCP::Venus) or RshWSCP (RshWSCP::Venus). We detected fluorescence derived from the chimeric proteins using a fluorescence microscope system. In cotyledons, fluorescence derived from AtWSCP::Venus and RshWSCP::Venus was detected in spindle structures. The spindle structures altered their shape to a globular form under blue light excitation. In true leaves, the number of spindle structures was drastically reduced. These observations indicate that the spindle structure was the ER body.

**Conclusions:**

AtWSCP and RshWSCP have the potential for ER body targeting like BoWSCP and LvWSCP.

## Background

Chlorophyll (Chl) is a photosynthetic pigment. Most chlorophyll proteins playing a role in photosynthesis are membranous proteins, but highly hydrophilic Chl proteins called water-soluble Chl-binding proteins (WSCPs) have been isolated from various land plants of Chenopodiaceae, Amaranthaceae, Polygonaceae, and Brassicaceae [1]. WSCPs from Chenopodiaceae, Amaranthaceae, and Polygonaceae are photoconvertible, but WSCPs from Brassicaceae do not show this ability [1]. Generally, photoconvertible and non-photoconvertible WSCPs are called Class I and Class II WSCPs, respectively [1]. Furthermore, Class II WSCPs are categorized into two subclasses, IIA and IIB, based on their Chl *a*/*b* ratio [1]. To date, *Lepidium virginicum* WSCP (LvWSCP) is the only Class IIB WSCP [1]. Chenopodiaceae WSCPs are members of the domain unknown function 538 superfamily and thus the biological function of these proteins remains unclear [2, 3]. On the other hand, all Class II WSCPs cloned thus far are members of the Kunitz-type trypsin inhibitor family [4–8]. The protease inhibitor activity of Class II WSCPs in young leaves was reported to be important for nitrogen remobilization under stressful conditions [9]. Class II WSCPs are also able to repress reactive oxygen species generation derived from excited Chl [10]. Additionally, *Brassica oleracea* WSCP (BoWSCP) and *Lepidium virginicum* WSCP (LvWSCP) are located in the endoplasmic reticulum (ER) body [6, 8], which is contributes to the a unique defense system in Brassicaceae [11, 12]. Thus, Class II WSCPs have the potential to act as Chl scavengers during cell disruption to protect healthy cells [6, 8]. Because the molecular structure of Class II WSCPs is quite simple and the complex is quite stable and easy to handle, Class II WSCPs are used as model proteins for characterizing the Chl–Chl and Chl–protein interactions of Chl proteins [13, 14].

BoWSCP and LvWSCP are highly accumulated in leaves and stems [15, 16], while *Arabidopsis thaliana* WSCP (AtWSCP) is expressed in the transmitting tract [5]. Moreover, *Raphanus sativus* var. *raphanistroides* Makino WSCP (RshWSCP) is highly transcribed in floral organs [7]. In contrast to BoWSCP and LvWSCP, the subcellular localization of AtWSCP and RshWSCP is still unclear. Elucidation of the subcellular localization of these proteins will provide clues for understanding the diversity and universality of Class II WSCPs.

## Methods

### Plant materials

*Arabidopsis thaliana* (ecotype, Col-0) was grown on 1 % agar plates containing full-strength MS medium and 1 % sucrose at 22 °C under continuous light. Three-week-old *A*. *thaliana* was transferred from the plate to Jiffy-7 for further growth.

### Isolation of genomic DNA

Using a DNeasy mini kit (Qiagen, Venlo, The Netherlands), we extracted and purified genomic DNA from leaves of 2-week-old *A*. *thaliana* according to the manufacturer’s instructions.

### Construction of transgenic *A*. *thaliana*

**Fig. 1:**
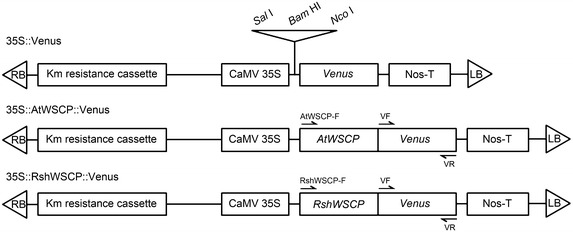
Schematic image of the control and modified Venus constructs for subcellular localization analysis of *Arabidopsis thaliana* WSCP (AtWSCP) and *Raphanus sativus* WSCP (RshWSCP)

The full-length regions of *AtWSCP* and *RshWSCP* were amplified by PCR with specific primer pairs containing a restriction enzyme site: for *AtWSCP*, 5′-GTCGACATGAAGAATCCTTCAGTGATCTCTTTTC-3′ and 5′-CCATGGAACCCGGGAAGTATAAGTTGCTAGTAGC-3′; for *RshWSCP*, 5′-GTCGACATGAAGAAACCTTCAGTGACCCCT-3′ and 5′-GGATCCGTAGAATGGGAACATCCTCAGACC-3′. Note that we used the RshWSCP::pGEM-T easy vector constructed in our previous study for RshWSCP amplification [7]. The PCR products were cloned into the pGEM-T easy vector (Promega). After sequence analysis, the correct clones were digested (*Sal*I and *Nco*I for the AtWSCP construct, *Sal*I and *Bam*HI for the RshWSCP construct) and then the DNA fragments were ligated into the same sites between the cauliflower mosaic virus (CaMV) 35S promoter and *Venus* in a modified pBI101 vector constructed in our previous study. All constructs used to generate transgenic plants are shown in Fig. 1. We introduced the constructs into *A*. *thaliana* using the floral dip method developed by Clough and Bent [17]. Transgenic plants were selected on 1 % agar plates containing 1 % sucrose, MS medium, 50 µg ml^−1^ kanamycin and 250 µg ml^−1^ cefotaxime sodium salt. Transformation of genes into *A*. *thaliana* was confirmed by PCR analysis with specific primer pairs (Fig. 1). The sequences of the primers used to detect *Venus* (i.e., VF and VR) and *Actin8* were described in our previous manuscript [8].

### Fluorescence microscopic analysis

To obtain fluorescence images of *A*. *thaliana* expressing Venus-fused chimeric AtWSCP and RshWSCP (i.e., AtWSCP::Venus and RshWSCP::Venus, respectively), we used an Axioskop2 plus microscope (Carl Zeiss) and Axio Cam (Carl Zeiss) with the Axio Vision software (Carl Zeiss). The T_2_ generations of transgenic plants were used for the fluorescence microscopic analysis.

## Results and discussion

**Fig. 2:**
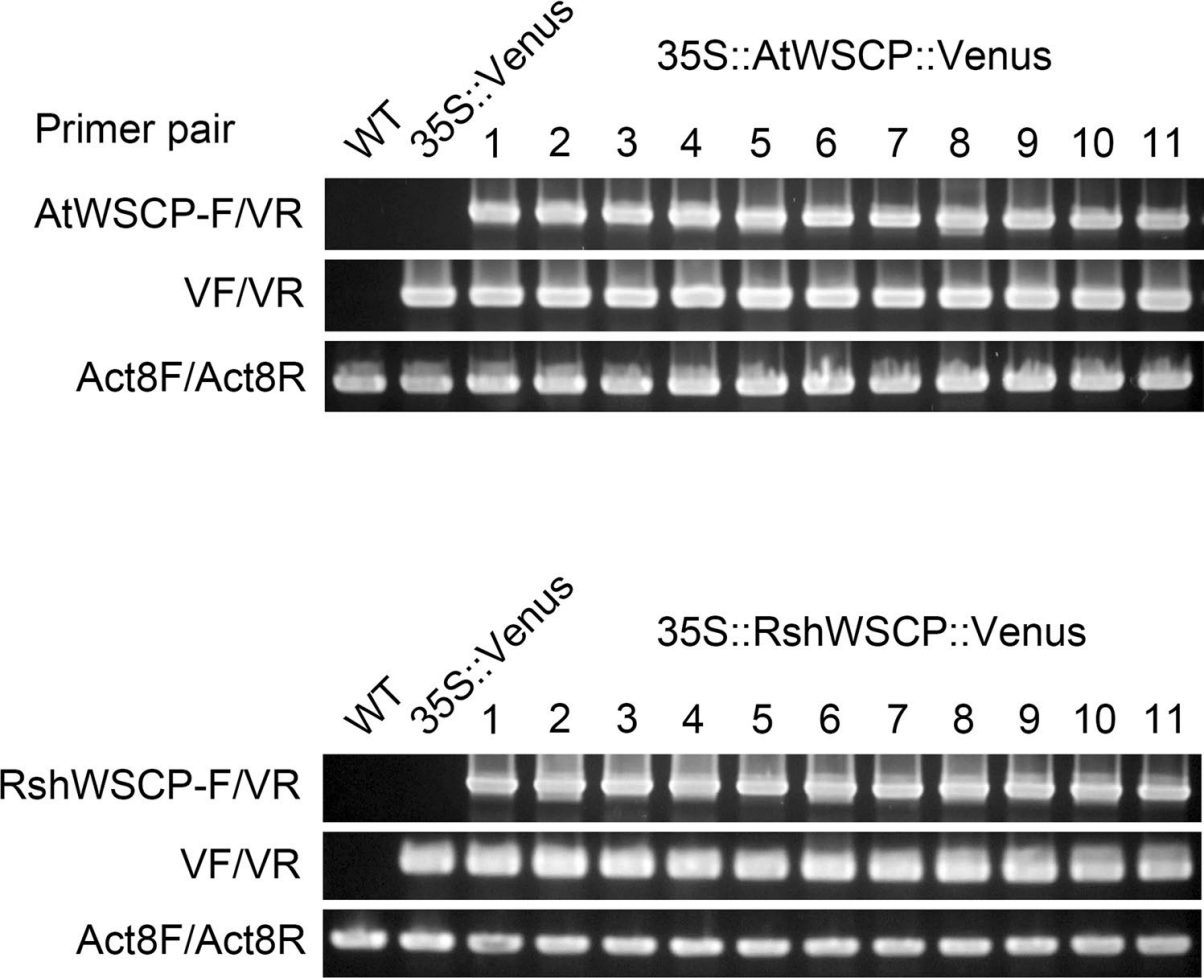
PCR analysis to confirm the correct insertion of cauliflower mosaic virus 35S promoter (35S)::AtWSCP::Venus and 35S::RshWSCP::Venus into *A*. *thaliana*. Primer sites (AtWSCP-F, RshWSCP-F, VF, and VR) are shown in Fig. 1. *Actin8* was used as a control

The aim of this study was to reveal the subcellular localization of AtWSCP and RshWSCP. In previous studies, we characterized the subcellular localization of BoWSCP and LvWSCP by analyzing transgenic *A*. *thaliana* expressing Venus-fused chimeric BoWSCP or LvWSCP (i.e., BoWSCP::Venus or LvWSCP::Venus) and found that both chimeric proteins were located in ER bodies [6, 8]. Here, we constructed transgenic AtWSCP::Venus or RshWSCP::Venus. Similar to BoWSCP and LvWSCP, both AtWSCP and RshWSCP possess an N-terminal extension, which is predicted to be a signal peptide. Thus, we introduced Venus at the C-termini of AtWSCP and RshWSCP. All constructs used to generate transgenic *A*. *thaliana* are shown in Fig. 1. The correctness of the transformants (35S::AtWSCP::Venus and 35S::RshWSCP::Venus) was confirmed by PCR analysis with the primer sets AtWSCP-F or RshWSCP-F/VR and VF/VR (Fig. 1). As shown in Fig. 2, we obtained a number of transformant lines.

**Fig. 3:**
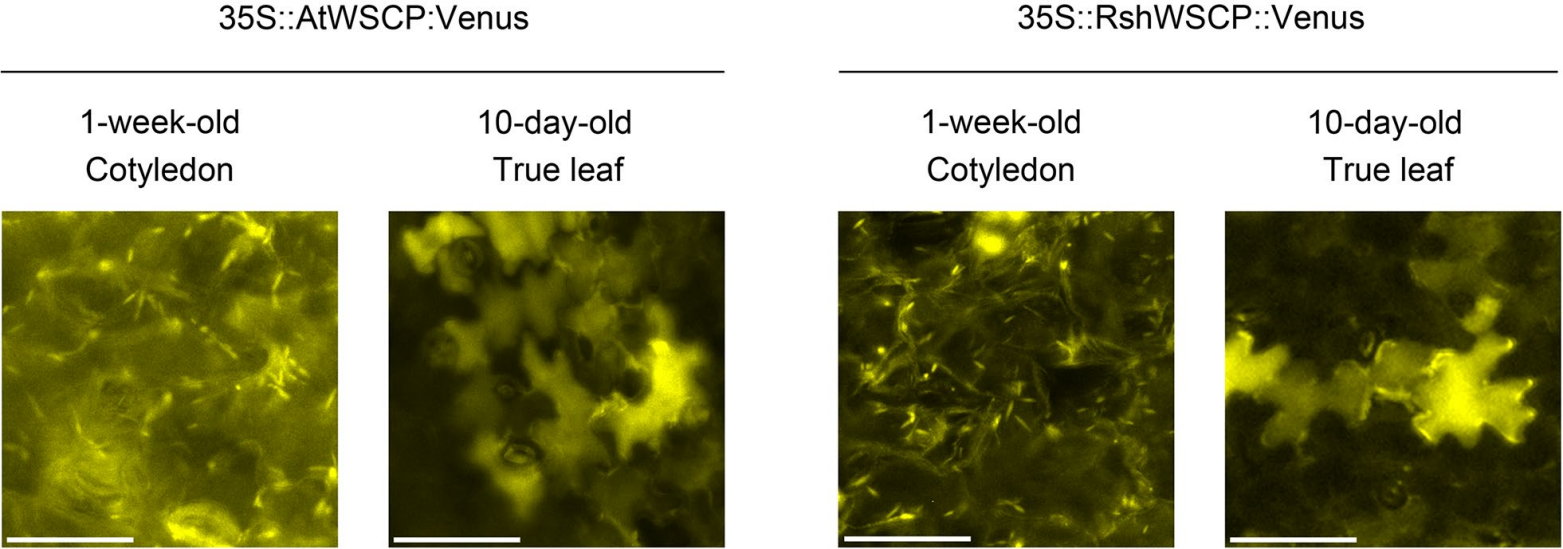
Fluorescence images of the transgenic plants. One-week-old cotyledons and 10-day-old true leaves of the T_2_ generation of transformants (35S::AtWSCP::Venus and 35S::RshWSCP::Venus), which were grown on 1 % agar plates containing full-strength Murashige and Skoog medium and 1 % sucrose at 22 °C under continuous light, were analyzed by fluorescence microscopy. The chimeric proteins were excited by blue light and then the fluorescence images were captured. *Bar* 20 µm

**Fig. 4:**
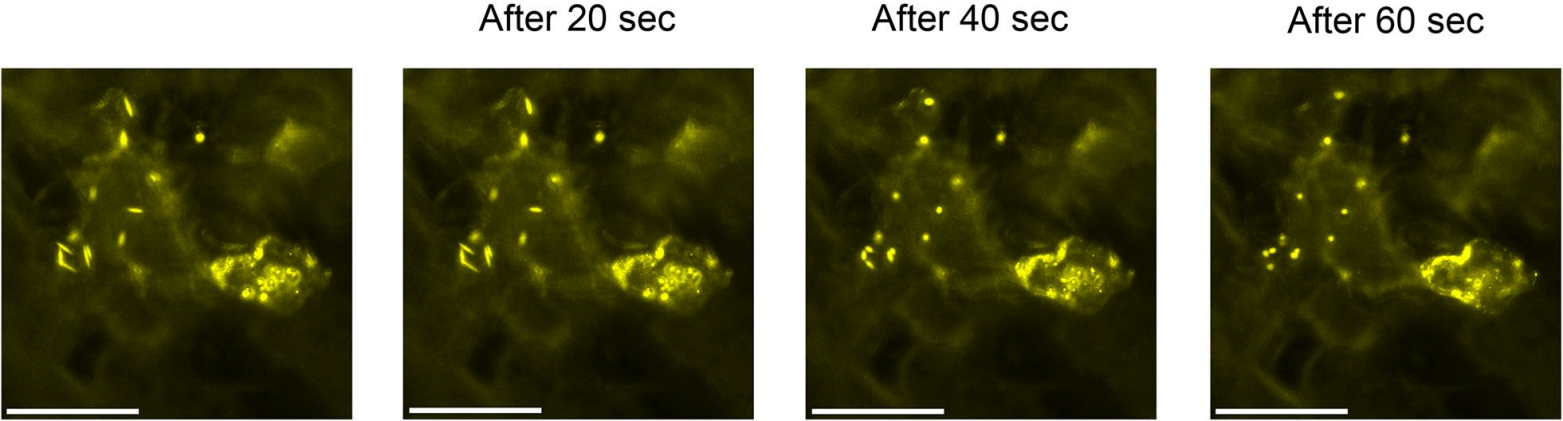
Time-dependent changes of the shape of the endoplasmic reticulum bodies. Fluorescence from 1-week-old cotyledons of 35S::RshWSCP::Venus was captured. *Bar* 20 µm

Figure 3 shows fluorescence images of leaves at different developmental stages from the transformants. In 1-week-old cotyledons of 35S::AtWSCP::Venus and 35S::RshWSCP::Venus plants, the fluorescence from Venus was detected in spindle-shaped structures. In the true leaves, however, most of these structures had disappeared and Venus fluorescence was instead detected in the vacuole. Furthermore, the spindle-shaped structures changed shape from spindle to globular during the fluorescence microscopic analysis with the high irradiation of blue light (Fig. 4). Brassicaceae plants have a unique spindle-shaped organelle called the ER body [11]. It was reported that the ER body changes its structure to a globular form and then fuses with the vacuole under high salinity conditions [18]. Recently, Gotté et al. reported that *Raphanus sativus* also has the ER body and that its shape was changed by methyl jasmonate treatment [19]. We therefore concluded that AtWSCP and RshWSCP can potentially target the ER body and the vacuole like BoWSCP and LvWSCP, but they do not target chloroplasts.

Because AtWSCP has protease inhibitor activity [20] and accumulates in the transmitting tract in the gynoecium and silique [5], Becktas et al. hypothesized that the protease inhibitor activity of AtWSCP might be important for the formation of the transmitting tract. Note that we could not find any difference between the transformants and wild type *A. thaliana*. Further analysis of the AtWSCP null-mutant will provide clues to elucidate the biological function of AtWSCP.

## Conclusion

To our knowledge, this is the first report describing the subcellular localization of floral organ-expressed WSCPs (i.e., AtWSCP and RshWSCP). Similar to leaf-expressed WSCPs (i.e., BoWSCP and LvWSCP), AtWSCP and RshWSCP target the ER body.

## Abbreviations

Chl: chlorophyll
ER: endoplasmic reticulum
WSCP: water-soluble chlorophyll-binding proteins
